# The autophagy *GABARAPL1* gene is epigenetically regulated in breast cancer models

**DOI:** 10.1186/s12885-015-1761-4

**Published:** 2015-10-17

**Authors:** Eric Hervouet, Aurore Claude-Taupin, Thierry Gauthier, Valérie Perez, Annick Fraichard, Pascale Adami, Gilles Despouy, Franck Monnien, Marie-Paule Algros, Michèle Jouvenot, Régis Delage-Mourroux, Michaël Boyer-Guittaut

**Affiliations:** 1 Université de Franche-Comté, Laboratoire de Biochimie, EA3922 « Estrogènes, Expression Génique et Pathologies du Système Nerveux Central », SFR IBCT FED4234, UFR Sciences et Techniques, 16 route de Gray, 25030 Besançon Cedex, France; 2 Department of Pathology, University Hospital Jean-Minjoz, 25030 Besançon, France

**Keywords:** Autophagy, GABARAP, GABARAPL1, GABARAPL2, Breast cancer, CREB-1, DNA methylation, Epigenetics

## Abstract

**Background:**

The GABARAP family members (GABARAP, GABARAPL1/GEC1 and GABARAPL2 /GATE-16) are involved in the intracellular transport of receptors and the autophagy pathway. We previously reported that *GABARAPL1* expression was frequently downregulated in cancer cells while a high *GABARAPL1* expression is a good prognosis marker for patients with lymph node-positive breast cancer.

**Methods:**

In this study, we asked using qRT-PCR, western blotting and epigenetic quantification whether the expression of the *GABARAP* family was regulated in breast cancer by epigenetic modifications.

**Results:**

Our data demonstrated that a specific decrease of *GABARAPL1* expression in breast cancers was associated with both DNA methylation and histone deacetylation and that CREB-1 recruitment on *GABARAPL1* promoter was required for *GABARAPL1* expression.

**Conclusions:**

Our work strongly suggests that epigenetic inhibitors and CREB-1 modulators may be used in the future to regulate autophagy in breast cancer cells.

**Electronic supplementary material:**

The online version of this article (doi:10.1186/s12885-015-1761-4) contains supplementary material, which is available to authorized users.

## Background

Autophagy is a cell process which regulates cell homeostasis and survival by inducing the degradation and recycling of intracellular components like protein aggregates or organelles (such as damaged mitochondria) [1]. This mechanism involves more than 40 proteins required for : i) the formation of autophagosomes, ii) the fusion of autophagosomes with lysosomes and/or, iii) the regulation of autophagy flux. The role of autophagy in tumorigenesis is controversial, even though evidence indicated that autophagy is often downregulated in cancer cells. On one hand, autophagy might protect against transformation of non tumoral cells into cancer cells and in the other hand, a loss of autophagy might help the cancer cells to escape from type II cell death (also called autophagy cell death) [2, 3]. Since induction of autophagy in cancer cells has also been described to confer resistance to chemotherapeutive agents, the co-administration of both alkyling agents and autophagy inhibitors (such as hydroxychloroquine) could improve the anti-tumoral response in these resistant cells [4]. Among the proteins involved in autophagy, two subfamilies of homologs of the yeast Atg8 (Autophagy-related 8) have been described in mammals: i) the MAP-LC3s (Microtubule-associated protein Light Chain 3) including LC3A, LC3B and LC3C and, ii) the GABARAP (GABA_A_-receptor-associated protein) family. The latter comprises 3 members: GABARAP, GABARAPL1/GEC1/ATG8L (GABARAP-like protein 1/guinea-pig endometrial glandular epithelial cells-1/Atg8-like protein) and GABARAPL2/GATE-16 (GABARAP-like protein 2/Golgi-associated ATPase enhancer of 16 kDa).

*GABARAP*, *GABARAPL1* and *GABARAPL2* genes are located on the human chromosomes, 17p13.12, 12p12.3 and 16q22.3 respectively, and are differentially expressed in normal and pathological tissues. The *GABARAP* gene has been described to be highly expressed in endocrine tissues while the *GABARAPL1* gene is predominantly expressed in the central nervous system but they both are underexpressed in a large variety of cancer cell lines [5]. Nevertheless, the analysis of *GABARAPL1* expression in a cohort of 256 breast adenocarcinoma revealed that a low *GABARAPL1* expression was correlated with a high risk of metastasis, in particular for lymph node-positive patients [6]. Despite these recent studies, the regulation of the *GABARAP* family is still poorly understood and the origin of their decreased expression in tumor models remains unknown.

It is now recognized that epigenetic modifications control the expression of numerous genes *via* the regulation of promoter accessibility to transcriptional factors. Both DNA methylation and histone modifications affect the level of chromatin compaction and it has been described that epigenetically-mediated aberrant silencing of genes are an important factor in the pathogenesis of cancers including breast cancers (BC) [7, 8] Indeed, epigenetic modifications can regulate the expression of a large panel of genes involved in the hallmarks of cancer, such as apoptosis, cell signaling, invasion and proliferation. For example, the detection of the promoter methylation of the tumor suppressor gene *BRCA1*, which is frequent in BC and is associated with a decrease of *BRCA1* expression, can help to predict the response to conventional chemotherapies in triple negative BC patients [9]. DNA methylation consists on the addition of a methyl group on a cytosine in CpG islands. It is catalyzed by DNA methyl transferases (DNMTs) and is unfavorable to transcription. Following DNA replication and formation of hemi-methylated DNA, the conservation of DNA methylation on the neo-synthesized strand, is mainly processed by DNMT1 using the parental strand as a model. This DNA methylation conservation is called maintaining or inheritance DNA methylation. On the opposite, *de novo* DNA methylation referred to the addition of DNA methylation on both strands of DNA on previously unmethylated loci is catalyzed by both DNMT3A and DNMT3B.

Besides DNA methylation, post-translational modifications of histones are also frequently associated to the regulation of gene expression in cancers. Histones are associated as octamers in nucleosomes (dimer of H2A, H2B, H3 and H4 and a loop of 126 pb DNA) whose compaction is regulated by post-translational modifications such as phosphorylation, methylation or acetylation. The local sum of these modifications is called the histone code and determine the status of local chromatin compaction (for a review see [10]). Histone methyl transferases (HMTs) or histone demethylases (HDMs) respectively catalyze the methylation or demethylation of histones leading to different effects on transcription. For example, H3K9me or K3K27me are negative marks while H3K4me is favorable to transcription. Acetylation, the most studied histone modification, is processed by histone acetyl transferases (HATs) and is associated with a local relaxed chromatin and is therefore favorable to gene expression. On the opposite, the removal of acetyl groups from histones, which is catalyzed by histone deacetylases (HDACs), contributes to gene silencing.

Some recent studies also revealed that epigenetic modifications can regulate autophagy gene expression as well as autophagy levels in both normal and cancer cells. For example, HDACs play an essential role in the regulation of autophagy: HDAC1 inhibition favors the conversion of the soluble LC3B form (LC3B-I) to the membrane-bound form of LC3B (LC3B-II), while the presence of H4K16ac (catalyzed by hMOF (human ortholog of drosophila males absent in the first)) in some *ATGs* genes, is associated to a decrease of expression of these genes [11, 12]. Moreover, HDAC6, an HDAC mainly localized in the cytosol, has also been described to be involved in the transport and maturation of autophagosomes [13]. DNA methylation is also involved in autophagy regulation as hypermethylation of several *ATG* genes has been described in various cancers [13, 14]. For example, methylation of *BECN*-*1*, a tumor suppressor gene, has been observed in BC, while methylation of *ATG16L2*, *LC3A*, *ULK2*, or *BNIP3* has been suggested to be involved in the down-regulation of autophagy in other cancers [15–19].

In order to characterize the regulation of *GABARAP* gene family expression in cancer cells, we analyzed their expression and the epigenetic modifications in the promoters of these genes in *in vitro* human BC cell models. Our data demonstrated that *GABARAPL1* expression is decreased in BC patients and BC cell line models. Moreover, both DNA methylation and deacetylation of histone H3 in *GABARAPL1* promoter were observed in these BC cell line models while the inhibition of DNMTs and HDACs using specific inhibitors restored *GABARAPL1* expression in these cells. These data suggest that DNMTi (DNMT inhibitors) and HDACi (HDAC inhibitors) could be used in the future to modulate autophagy levels in BC cells.

## Methods

### Ethic statement

Human samples were collected according to French laws and the recommendations of the French National Committee of Ethics. Indeed, this study has been approved by of the scientific committee of “the tumorothèque régionale de Franche-Comté BB-0033-00024”. The samples and the medical history of patients were encoded to protect patients confidentiality and used under protocols approved by the recommendations of the French national Committee of Ethics. All human samples were collected by Pr. Severine Valmary-Degano (Centre Hospitalo-Universitaire, Besançon, France) at the “Tumorothèque régionale de Franche-Comté BB-0033-00024”. Collection of samples and their use (AC-2010-1163) for studies (approved by the scientific committee of “the tumorothèque régionale de Franche-Comté BB-0033-00024”) have been approved by the French “ministère de la recherché” and by the CPP EST II.We obtained all necessary consents from any patients involved in the study.

### Quantitative RT-PCR

RNA was isolated from cells and frozen tissues using Tri Reagent (Molecular Research Center, TR-118) as described by the manufacturer. Reverse transcription were performed using M-MLV (Sigma-Aldrich, M-1302) reverse transcriptase and 1.5 μg total RNA according to manufacturer’s instructions (Sigma-Aldrich). Quantitative PCR (qPCR) were done in duplicate using the Step one Real-Time PCR system (Applied Biosystems), Power SYBR Green PCR Master Mix (Applied Biosystems, 4367659), according to manufacturer’s instructions and primers specific of *GABARAP* (F: 5′-GCCTTTCCCATCCTGCTGTA-3′ and R: 5′-AGGAGGGGATTGCTGGGTTCT-3′) ; *GABARAPL1* (F: 5′-CCCTCCCTTGGTTATCATCCA-3′ and R: 5′-ACTCCCACCCCACAAAATCC-3′) and *GABARAPL2* (F: 5′-AAATATCCCGACAGGGTTCC-3′ and R: 5′-CAGGAAGATCGCCTTTTCAG-3′). *H3B2* was used as an housekeeping gene (F: 5′-GCTAGCTGGATGTCTTTTGG-3′ and R: 5′-GTGGTAAAGCACCCAGGAA-3′) as previously described [20].

### Cell culture

MCF-7 and MDA-MB-453 cell lines were obtained from ATCC (HTB-130 and HTB-22) and grown in DMEM 1 g/L glucose (Dominique Dutscher, L0066) containing fetal calf serum (5 %) (Dominique Dutscher, S1810), penicillin (50 U/ml) (Dominique Dutscher, L0018), streptomycin (50 μg/ml), and amphotericin B (1.25 μg/ml) (PAA, P11-001) at 37 °C in 5 % CO_2_, and routinely used at 70–80 % confluence. When indicated, cells were exposed to 2 μM 5-aza-CdR (A3656, Sigma-Aldrich) for 48 h and 400 nM TSA (T8552, Sigma-Aldrich) for 16 h.

### Luciferase activity

*GABARAPL1* promoter fragments were obtained by PCR using the following primers–336 F: 5′-GCTGGATCCCAACCAGCAGGA-3′, −659 F: 5′-GTCAGGCTGGTCTCGAACTC-3′ and +241 R: 5′-GGGATGCACCGCAGGGCTTCC-3′ and then cloned into the pGL3 basic plasmid. 5000 cells were seeded in 96 multiwell dishes and cells were transfected with pGL3 plasmids, co-transfected with the pCDNA3.1-CREB-1 vector (kindly provided by Vincent Coulon, Montpellier, France) or treated with 10 μM forskolin. Luciferase expression was measured using the Luciferase Assay System kit (E1500, Promega) according to the manufacturer’s recommendations.

### Epigenetics

gDNA was extracted using the NucleoSpin® Tissue kit (740952, Macherey Nagel). Global DNA methylation was quantified using the MethylFlash methylated DNA quantification kit (P-1034, Epigentek, France). Methyl DNA collection were performed using the “Methyl-Collector Ultra kit” (55005, Active Motif). Histone 3 acetylation was quantified using the EpiQuik Tissue Acetyl-Histone H3 ChIP Kit (P-2012, Epigentek). ChIP was performed using the ChIP-IT High Sensitivity kit (53040, Active Motif) with ChIP grade antibodies (Table 1). All these kits were used according to the manufacturer’s instructions. Primers used in this study were designed with the primer3 software [21]: *GABARAP* (F: 5′-AAAGCCAACCGTCTTTGCTA-3′ and R: 5′-GCCACTTCCCTATTCACCAA-3′), *GABARAPL1* (MC1 F: 5′-GTCAGGCTGGTCTCGAACTC-3′ and R: 5′-CGCTCCTGAACAGCAACATA-3′) and *GABARAPL1* (MC2 F: 5′-AAGGAAACGCAGTGAGACAGA-3′ and R: 5′-AGCTGGGAGCACAAAAACAG-3′), *GABARAPL2* (F: 5′AATTCCCCAGACTTCCCCTA-3′ and R: 5′-GGTGGCGAAGAAGTTGGTTA-3′).

**Table 1:** List of antibodies

Antibody	Application	Dilution	Manufacturer	Ref
GABARAP/GABARAPL1	WB	1:3000	(Millipore, France)	#AB15278
GABARAPL2	WB	1:1000	(Proteintech, France)	#18727-AP
ACTIN	WB	1:3000	(Sigma-Aldrich, France)	#A5060
CREB-1	IF/ChIP	1:50/1 μg	(Santa-Cruz Biotechnology, France)	#sc-374227
DNMT1	ChIP	1 μg	(Active Motif, Belgium)	#39204
HDAC1	ChIP	1 μg	(Active Motif, Belgium)	#40967

### Western-blotting

Cells were scraped, harvested and lysed in RIPA buffer (50 mM Tris–HCl, pH 8, 150 mM NaCl, 1 % Triton ×100, 0.5 % DOCA, 0.1 % SDS) supplemented with protease inhibitors (104 mM AEBSF, 1.5 mM pepstatin A, 1.4 mM E-64, 4 mM bestatin, 2 mM leupeptin, 80 μM aprotinin) for 30 min on ice, sonicated for 15 sec and centrifuged at 10 000 g for 10 min at 4 °C. Supernatant was used for protein quantification using the Bradford method [22] and then proteins (40 μg) were separated using SDS-PAGE gels and transferred to PVDF membranes (Bio-Rad, 162–0177) for 2 h in Tris-Glycine buffer as previously described [23]. Membranes were saturated in 0.1 % TBS-Tween 20 and 5 % nonfat milk for 1 h and then incubated with primary antibodies (Table 1) overnight at 4 °C. Membranes were washed 3 times with TBS-Tween 20 0.1 %, incubated with secondary anti-rabbit HRP conjugate or anti-mouse HRP conjugate antibody according to manufacturer’s instructions (P.A.R.I.S., BI2407 and BI2413C). The membrane was washed 3 times with TBS-0.1 % Tween 20 incubated with ECL revelation buffer (Pierce) and cheluminescence was monitored using a ChemiDoc^TM^XRS+ (Biorad).

### Immunofluorescence

CREB-1 IF was performed as precognized by the manufacturer. Briefly, cells were seeded on coverslips in 24 multiwell-plates, fixed and permeabilized for 20 min with cold methanol at–20 °C, washed 3 times with cold PBS (Phosphate buffer saline : 137 mM NaCl, 2.7 mM KCl, 10 mM Na_2_HPO_4_, 2 mM KH_2_PO_4_), incubated with 1 % BSA-PBS for 1 h at 37 °C and incubated overnight with the CREB-1 antibody (Table 1). The coverslips were washed 3 times for 5 min with 0.1 % Tween-PBS (T-PBS), incubated for 1 h with a goat secondary anti-mouse Alexa 555 antibody (Life technology, France) and washed 3 times for 5 min with (T-PBS). Cells were then mounted in Vectashield Hardset mounting medium (Vector Laboratories, H-1000) and analyzed using an Olympus IX81 confocal microscope (Olympus, France).

### Statistics

Mean’s comparison were analyzed using a Student t-test with GraphPad Prism5 software (USA). Correlation indexes were measured using a Spearman test with ImageJ software. Significant values were highlighted in bold in each figure.

## Results

### *GABARAP* family genes are differentially expressed in human breast cancer biopsies

**Fig. 1:**
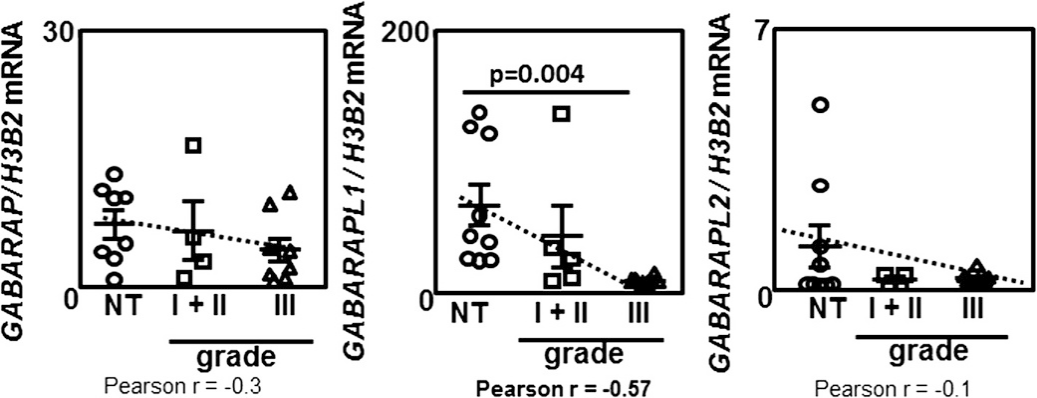
*GABARAP* family expression is deregulated in breast cancers. Quantification of *GABARAP*, *GABARAPL1*, *GABARAPL2* expression using qRT-PCR, in grade I + II and grade III BC biopsies compared to normal adjacent tissue. Circle: NT (non tumoral), square: grade II, triangle: grade III. Difference of expression were quantified using a t-test. The correlation between the tumor grade and gene expression was measured using a Spearman test

We first analyzed *GABARAP*, *GABARAPL1* or *GABARAPL2* mRNA expression in human BC biopsies using qRT-PCR (Fig. 1). The different BC subtypes are classified in regard of their molecular marker expression. Luminal BC, which represent 50 % of total BC and generally associated with a good prognosis, are divided in Luminal A and Luminal B BC. Both Luminal A and B BC express ERα (estrogen receptor: ER+) whereas the expression of the *HER2* (HER+) (human epidermal growth factor receptor) gene was only observed in Luminal B BC. HER+ BC subtype, which represents 17 % of BC, presents an amplification of the *HER2* gene without the expression of ERα (ER-). The triple negative BC (ER-/PR-/HER-) do not express ERα, PR (progesterone receptor) and HER2 and cannot be treated with specific therapies (for a review, see [24]). Our cohort comprised 5 grade I BC (ER+/PR+/HER-) and 8 grade III BC (5 ER-/PR-/HER-and 3 ER+/PR+/HER+). Our results revealed an insignificant decrease of both *GABARAP* and *GABARAPL2* mRNA levels in grade III BC compared to non tumoral tissue (NT). More interestingly, *GABARAPL1* expression was strongly decreased in BC grade III tissues (*p* = 0.004) versus non tumoral tissues. An inverse correlation (*r* = −0.57) was also observed between *GABARAPL1* mRNA and tumor stage while a very poor correlation was determined between *GABARAP* or *GABARAPL2* expression levels and the tumor grade (respectively *r* = −0.3 and *r* = −0.1).

### Study of global and local epigenetic modifications in *GABARAPL1* promoter and *GABARAPL1* expression in BC tissues and cell models

**Fig. 2:**
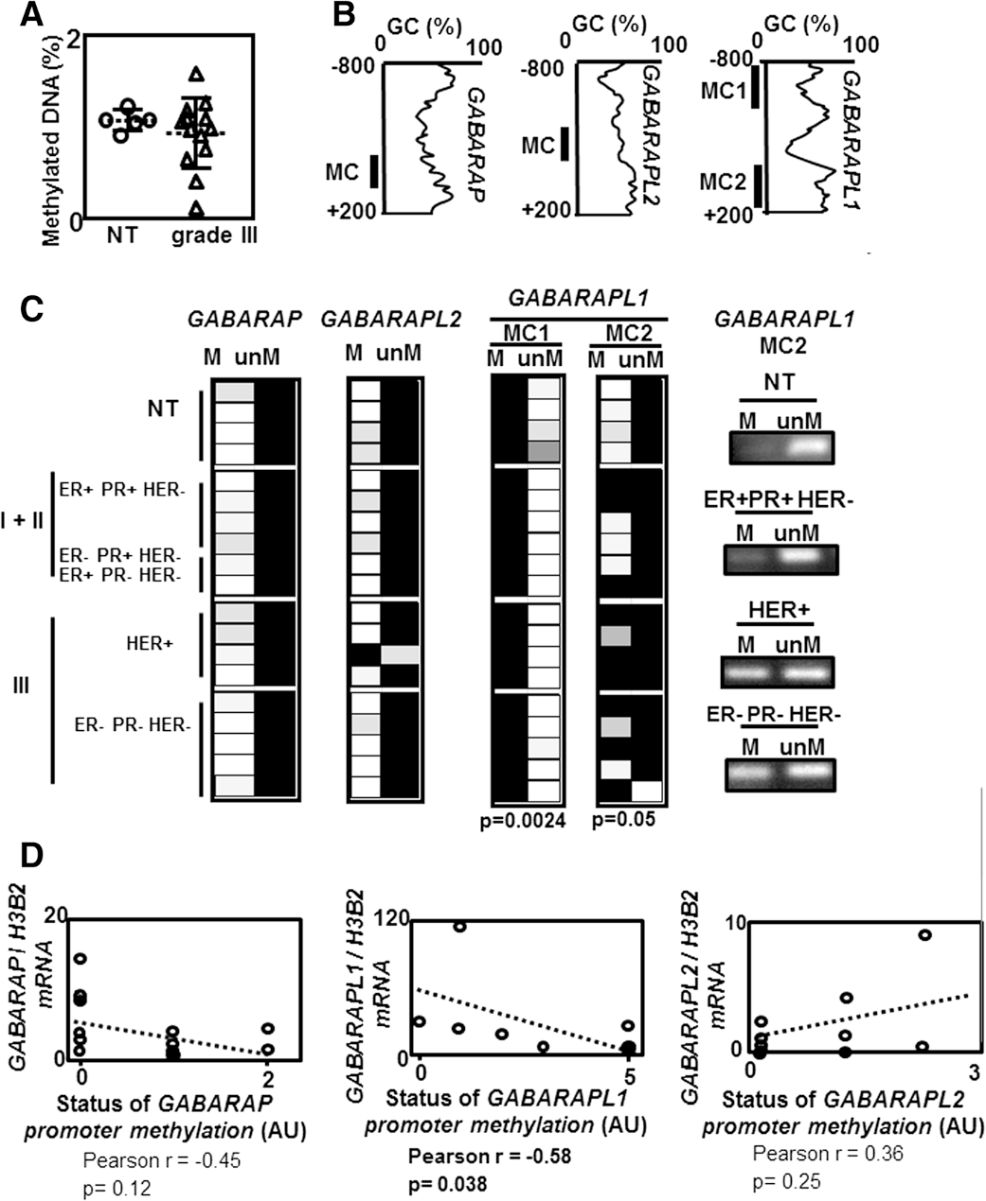
Detection of DNA methylation in *GABARAP* family promoters **a** Global DNA methylation quantification in BC and non tumoral biopsies using methylFlash ELISA kit. **b** Scheme describing the *GABARAP* family gene promoter structure (Methprimer) and primer localization. **c** Left: Descriptive of *GABARAP* family gene methylation using methylCollector kit ; right : examples of methylation signal observed using *GABARAPL1* MC2 primers. White : absence of signal of methylation; black : signal of methylation. **d** Correlations (Spearman test) between expression and methylation of *GABARAP*, *GABARAPL1* and *GABARAPL2* genes. NT: non tumoral, ER +/−: status of expression of estrogen receptor α, PR+/−: status of expression of progesterone receptor, HER+/−: status of expression of Human epidermal growth factor receptor; I-III: BC grade

Since tumors are frequently associated with aberrant DNA methylation content, we quantified global DNA methylation levels using ELISA in gDNA issued from both grade III BC samples (associated with the lowest expression of *GABARAPL1* mRNA, as previously described in Fig. 1 and NT tissues (Fig. 2a).^7^ [25, 26] As expected, several gDNA issued from grade III BC presented a lower global DNA methylation compared to gDNA issued from NT tissues. These results suggested that epigenetic modifications might occur in these tumors. To determine whether local DNA methylation was also altered in these BC samples, methylation of *XIST*, a X-linked gene known to be methylated in women, and *GAPDH*, a gene constitutively active and unmethylated, were analyzed by precipitation of methylated DNA using the methylCollector Ultra kit. As expected, methylation of *XIST* was observed in 100 % of both gDNA issued from NT and grade I-II BC tissues tested but lost in 3 out of 5 triple negative BC biopsies (Grade III) (Additional file 1: Figure S1). On the contrary, *GAPDH* was never found to be methylated in NT gDNA but was surprisingly frequently methylated in gDNA issued from BC samples (Additional file 1: Figure S1). Loss of global DNA methylation in several tissues (Fig. 2a 4 out 13 samples) and aberrant status of methylation of *XIST* and *GAPDH* (Additional file 1: Figure S1) strongly suggested that DNA methylation was deregulated in BC samples.

We next analyzed whether epigenetic regulation of the *GABARAPL1* might explain its specific down-regulation in BC. *GABARAP*, *GABARAPL1* and *GABARAPL2* present CpG rich areas in their promoter (−800/+200) as predicted using the methPrimer software [27], so we analyzed the methylation status of these promoters using the methylCollector Ultra kit (Fig. 2b and c). A low methylation signal was observed in both *GABARAP* and *GABARAPL2* promoters (MC primers) both in tumors and NT tissues while primers designed in the–800 region of *GABARAPL1* (MC1) (Fig. 2c) revealed a strong signal of methylation in both NT and BC tissues. Regarding *GABARAPL1*, a low signal of unmethylation was also measured in NT samples but not in BC samples suggesting that hemi-methylation was lost in cancer cells (*p* = 0.0024) (Fig. 2c). To confirm the higher level of methylation in the *GABARAPL1* promoter in BC, the same experiment was repeated using primers (MC2) designed to detect the 5′-UTR region of this gene. Results obtained with MC2 primers showed that *GABARAPL1* was not or weakly methylated in NT samples but highly methylated in BC tissues (*p* = 0.05) (Fig. 2c). Pearson correlation analysis revealed that *GABARAP* or *GABARAPL2* expression was not correlated with methylation status (r = −0.45, *p* = 0.12 and r = 0.36, *p* = 0.25 Fig. 2d), while *GABARAPL1* expression was indeed correlated with the methylation status of the gene (r = −0.58, *p* = 0.03 suggesting that methylation of the *GABARAPL1* promoter may explain the downregulation of *GABARAPL1* expression in BC (Fig. 2d).

**Fig. 3:**
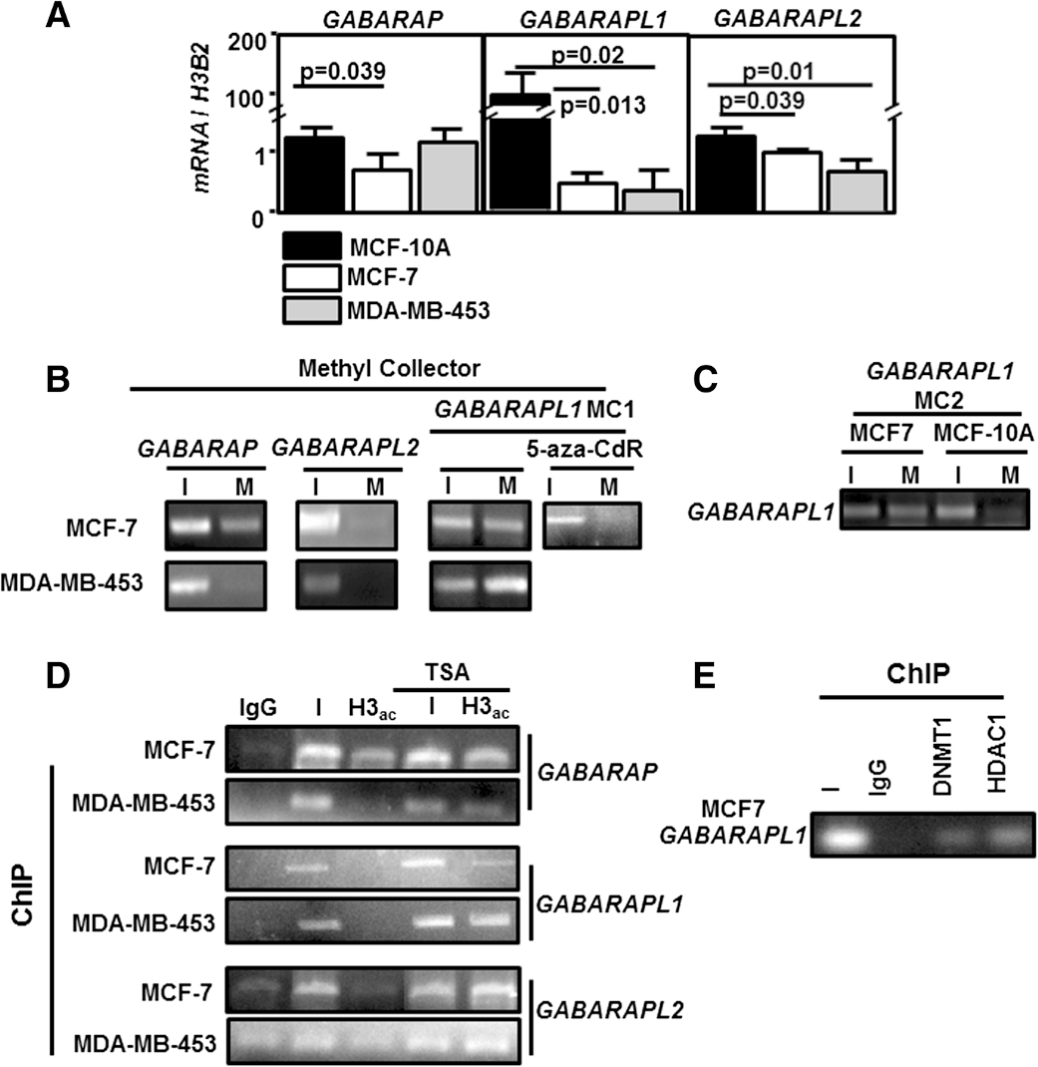
Epigenetic modifications in *GABARAP* family gene promoters. **a** Quantification of *GABARAP*, *GABARAPL1*, *GABARAPL2* expression using qRT-PCR in BC MCF-7 and MDA-MB-453 cancer cells and MCF-10A immortalized cells. **b** and **c** *GABARAP* family gene methylation using methylCollector kit in MCF-7, MDA-MB-453 and MCF-10A cells. (I: input; M: methylated fraction). **d** Visualization of H3 deacetylation using ChIP experiment and anti-H3 acetylated (H3-ac) antibody in the *GABARAP* family gene in MCF-7 and MDA-MB-453 cells (I: input; IgG: negative control of IP). **e** Detection of DNMT1 and HDAC1 recruitment on *GABARAPL1* promoter using ChIP experiment and anti-DNMT1 or anti-HDAC1 antibody (I: input; IgG : negative control)

In order to characterize the pathway allowing epigenetic modifications to control *GABARAP* family expression, we first analyzed *GABARAP*, *GABARAPL1* and *GABARAPL2* mRNA levels using qRT-PCR in MCF-7 and MDA-MB-453 (BC cell lines) and MCF-10A (Breast immortalized but non tumoral cell line) cell lines (Fig. 3a). Both *GABARAP* (*p* = 0.039), *GABARAPL1* (*p* = 0.013) and *GABARAPL2* (*p* = 0.039) expression were decreased in MCF-7 compared to MCF-10A but while the loss of *GABARAP* or *GABARAPL2* expression was weak (0.5-2 fold), *GABARAPL1* expression was about 100-fold lower in MCF-7 compared to MCF-10A (Fig. 3a). Similar results were obtained for *GABARAP*, *GABARAPL1* and *GABARAPL2* in MDA-MB-453 cells but *GABARAP* did not show any significant differences in this cell line (Fig. 3a). Methylation of the *GABARAP* family promoters was then assessed using the MethylCollector kit in both MCF-7 and MDA-MB-453 cell lines presenting a low expression of *GABARAPL1*. First, a signal of methylation of *GABARAP* promoter was observed in MCF-7 cells but not in MDA-MB-453 cells (Fig. 3b). Regarding *GABARAPL2* promoter, no methylation was detected in both cell lines. A high signal of methylation was detected in the *GABARAPL1* promoter in MCF-7 and MDA-MB-453 cells using MC1 primers confirming that the region of MC1 is highly frequently methylated. As expected *GABARAPL1* promoter methylation was lost in MCF-7 cells treated with 5-aza-CdRdeoxycytidine (5-aza-CdR), a DNMTi (Fig. 3b). A high signal of methylation was also detected using MC2 primers in MCF-7 cells but not in MCF-10A cells suggesting, as observed before in human BC biopsies, that *GABARAPL1* methylation is predominantly observed in BC cell lines (Fig. 3c)

As local DNA methylation is frequently associated to histone deacetylation, the acetylation status of histone H3 (H3-ac) was analyzed by ChIP in MCF-7 and MDA-MB-453 cells previously treated or not with trichostatin A (TSA), an inhibitor of HDACs (Fig. 3d). ChIP analysis revealed that H3 acetylation was detected in the *GABARAP* promoter in MCF-7 cells but not in MDA-MB-453 cells. A very low level of H3 acetylation was also observed in *GABARAPL2* promoter in these both cell lines. These signals were increased following TSA treatment in MCF-7 and MDA-MB-453 cells. Similarly, no H3 acetylation signal could be detected in the *GABARAPL1* promoter of BC cells but this signal was increased after TSA treatment, particularly in the MDA-MB-453 cells (Fig. 3d).

All these data (Figs. 1, 2 and 3) suggest that *GABARAPL1* is the most regulated gene of the *GABARAP* family and that require promoter deacetylation and DNA methylation. Interestingly, both DNMT1, which predominantly catalyzes inheritance DNA methylation, and HDAC1 were detected on *GABARAPL1* promoter in MCF-7 cells using ChIP experiments (Fig. 3e).

**Fig. 4:**
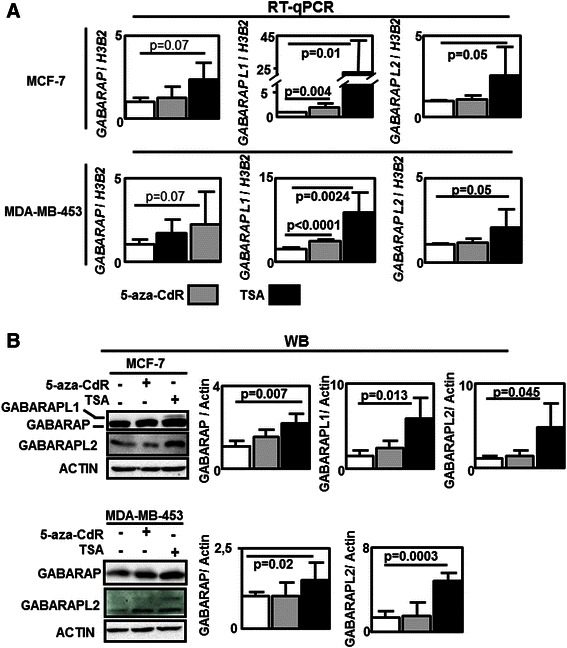
Effects of epigenetic modulators on the *GABARAP* family gene expression. **a** Effects of 5-aza-deoxycytidine (5-aza-CdR) or trichostatin A (TSA) on *GABARAP* family gene expression using qRT-PCR analysis. **b** Effects of 5-aza-CdR or TSA on GABARAP family protein expression using WB (anti-GABARAP/GABARAPL1, anti-GABARAPL2 and anti-ACTIN antibodies) in cells previously treated with MG-132. Differences were quantified using a t-test

We next asked whether 5-aza-CdR or TSA could restore *GABARAPL1* expression in BC cell lines. To do so, MCF-7 and MDA-MB-453 cells were treated with 5-aza-CdR or TSA and the levels of *GABARAP*, *GABARAPL1* and *GABARAPL2* mRNA were measured using qRT-PCR (Fig. 4a) while protein levels were quantified by western-blotting (Fig. 4b). All cells were also treated with MG-132, before protein extraction, to prevent the fast proteasomal degradation of GABARAPL1 which has been previously reported [28]. First we observed a not significant increase of *GABARAP* mRNA (*p* = 0.07) but a significant increase of the corresponding protein GABARAP (*p* = 0.007 and *p* = 0.02) following TSA treatment in MCF-7 and MDA-MB-453 cells. Both *GABARAPL2* mRNA (*p* = 0.05 and *p* = 0.05 respectively) and GABARAPL2 protein (*p* = 0.045 and *p* = 0.0003 respectively) were increased in MCF-7 and MDA-MB-453 cells treated with TSA. No significant effect of 5-aza-CdR could be observed on *GABARAP* and *GABARAPL2* expression (Fig. 4a). On the opposite, 5-aza-CdR treatment increased *GABARAPL1* mRNA level (about 2 fold) in both cell lines (*p* = 0.004 and *p* < 0.0001 respectively) while TSA treatment increased *GABARAPL1* expression of 10 to 25 fold (Fig. 4a). Moreover, GABARAPL1 expression, which was undetectable in non treated MCF-7 cells or 5-aza-CdR-treated cells, was increased following TSA treatment (Fig. 4b). A weak and diffuse band signal corresponding to the GABARAPL1 protein was also in over-exposed western-blotting using lysates of MDA-MB-453 cells treated with TSA, suggesting that GABARAPL1 might also be slightly increased in these cells after TSA treatment.

Altogether these results confirm that *GABARAPL1* is the gene of the *GABARAP* family whose expression is the most sensitive to epigenetic regulation in BC cell lines. Since 5-aza-CdR and TSA treatments restored GABARAPL1 content, we next asked whether these compounds modulate autophagy and cell proliferation in MCF-7 cells (Additional file 2: Figure S2). Both an increase of LC3B-II (form associated to the autophagosomes) (Additional file 2: Figure S2A) and of cells with GFP-LC3 puncta (Additional file 2: Figure S2B) were observed in respectively 5-aza-CdR/ TSA treated cells and in GFP-LC3 transfected and TSA treated cells compared to control cells. Moreover, both an decrease of cell proliferation and clonogenecity were also observed in MCF-7 treated cells (Additional file 2: Figure S2C and D). These results suggest that restoration of GABARAPL1 expression might be linked to these processes although some pleiotropic effects of 5-aza-CdR and TSA treatment could also be involved [29].

### *GABARAPL1* expression is regulated by CREB-1

**Fig. 5:**
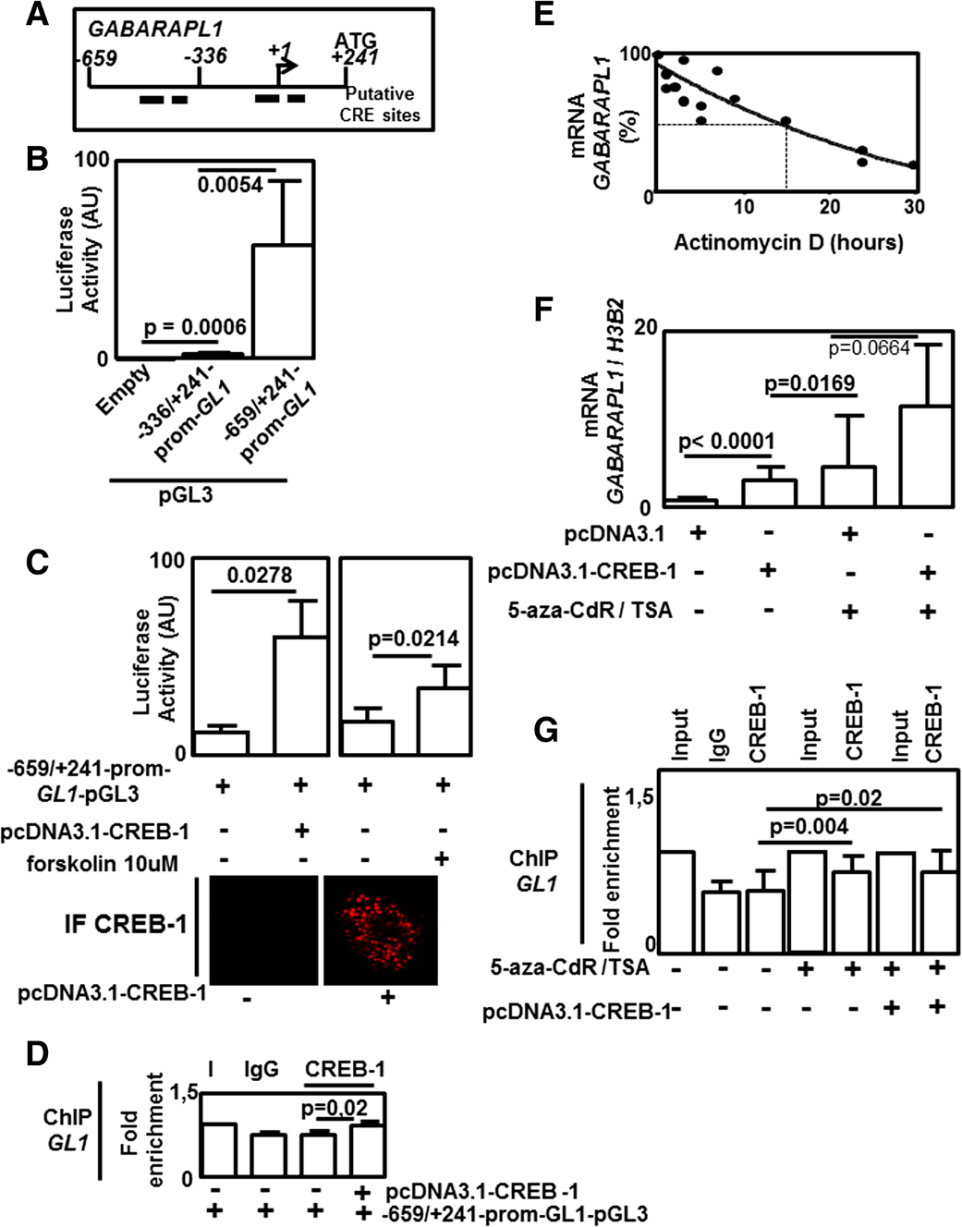
*GABARAPL1* expression I controlled by CREB-1. **a** Scheme describing the position of primers used (in regard of the putative initial transcription site (+1)) and putative CRE (CREB-1 response elements) sites in the *GABARAPL1* promoter. **b** and **c** Luciferase activity measured using a Luciferase assay System Kit in MCF-7 cells transfected with empty pGL3 plasmid,−336/+241-*GABARAPL1*-promoter-pGL3 plasmid,–659/+241-*GABARAPL1*-promoter-pGL3 plasmid, pCDNA3.1-CREB-1 or treated with (10 μM) forskolin. **c** Bottom : expression of CREB-1 using IF in cells transfected or not with the pCDN3A.1-CREB-1 vector. **d** Recruitment of CREB-1 on–659/+241-*GABARAPL1*-promoter-pGL3 plasmid using ChIP experiment and anti-CREB-1 antibody in MCF-7 cells transfected with–659/+241-*GABARAPL1*-promoter-pGL3 and pCDNA3.1-CREB-1 plasmids (I: input; IgG : negative control of IP). **e** Half-life of *GABARAPL1* mRNA using qRT-PCR following Actinomycin D treatment in MCF-7 cells. **f** Effects of CREB-1 overexpression (following pCDNA3.1-CREB-1 plasmid transfection) and/or 5-aza-CdR /TSA treatment on *GABARAPL1* expression using qRT-PCR in MCF-7 cells. **g** Effects of CREB-1 overexpression (following pCDNA3.1-CREB-1 plasmid transfection) and/or 5-aza-CdR/TSA treatment on CREB-1 recruitment in *GABARAPL1* promoter using ChIP experiment and an anti-CREB-1 antibody (I: input; IgG : negative control). Differences were quantified using a t-tests. GL1: GABARAPL1

Behind epigenetic modifications of the promoter, the second step of gene regulation is the recruitment of transcriptional factors (TF). Therefore, in order to characterize the mechanisms governing *GABARAPL1* expression in these cells, we cloned two fragments of the *GABARAPL1* promoter (−659/+241 and −336/+241) in the pGL3 luciferase reporter plasmid. As suggested by 3 different softwares (TESS http://www.cbil.upenn.edu/tess, Cister http://zlab.bu.edu/~mfrith/cister.shtml and Patch http://www.gene-regulation.com/pub/programs.html#patch) used to predict TF binding sites, several putative CRE (cAMP Response Element) elements were identified in *GABARAPL1* promoter (Fig. 5a). Indeed, following transfection of our constructions in MCF-7 cells, we observed a significant increase of luciferase signal in cells transfected with the–336/+241-*GABARAPL1*-promoter-pGL3 plasmid compared to the empty pGL3 vector suggesting the presence of functional regulatory elements in this region. Moreover, luciferase activity was strongly increased when cells were transfected with the–659/+241-*GABARAPL1*–promoter-pGL3 vector compared to the basal–336/+241-*GABARAPL1*-promoter-pGL3 vector suggesting that the region −659/-336 is also important for *GABARAPL1* regulation (Fig. 5b). Both treatment of MCF-7 cells with forskolin, a compound known to activate CREB-1 (CRE binding protein-1), or transfection with a plasmid expressing the CREB-1 protein significantly increased luciferase activity linked to the construction–659/+241-*GABARAPL1*–promoter-pGL3, suggesting that CREB-1 is indeed involved in *GABARAPL1* expression (Fig. 5c). The increase of luciferase activity in cells transfected with the pcDNA3.1-CREB-1 vector might be explained by the low level of endogenous CREB-1 in MCF-7 cells as observed in IF experiments (Fig. 5c) and ChIP experiment also confirmed the recruitment of CREB-1 on the–659/+241-*GABARAPL1*-promoter in spite of the presence of a high background noise that may be provoked by the presence of–659/+241-*GABARAPL1*-promoter plasmid (Fig. 5d). We next asked whether endogenous *GABARAPL1* expression may be regulated by CREB-1. Treatment of MCF-7 cells with Actinomycin D revealed that the half-life of *GABARAPL1* mRNA was high (about 17 h) suggesting that regulation of *GABARAPL1* mRNA content is more dependent on transcription that mechanisms affecting mRNA stability (Fig. 5e) [30, 31]. Moreover, overexpression of CREB-1 in MCF-7 cells significantly increased *GABARAPL1* expression but at a lower level than the ones observed in cells treated with 5-aza-CdR/TSA (Fig. 5f). Moreover, a non-significant (*p* = 0.066) further increase of *GABARAPL1* expression was observed in cells transfected with the CREB-1 plasmid and treated with 5-aza-CdR/TSA compared to cells with 5-aza-CdR/TSA treatment alone.

These data suggest that epigenetic modifications may be an initial predominant factor allowing the recruitment of CREB-1 on *GABARAPL1* promoter. These observations were partly confirmed by ChIP experiments showing an increase of CREB-1 recruitment on *GABARAPL1* promoter in MCF-7 cells previously treated with 5-aza-CdR/TSA. But no significant difference could be observed between 5-aza-CdR/TSA treated cells transfected or not with the vector encoding CREB-1 (Fig. 5g).

## Discussion

While the role of autophagy in tumorigenesis is still controversial, it is currently admitted that autophagy is generally reduced in cancer cells. Whereas the origin of the autophagy disruption is still unknown, mutations or loss of heterozygosity-dependent autophagy gene silencing (*LC3*, *ATGs*, *UVRAG*, *BECN*-*1*) have been reported in many different cancers, such as colorectal, gastric carcinoma or breast cancers [32–36]. Besides mutations, epigenetic modifications frequently occur in cancers, such as global DNA hypomethylation and/or both local hypo and hypermethylations in specific loci [37–39]. Hypermethylation of five autophagy-related genes, *BECN*-*1*, *ATG16L2*, *ULK2*, *BNIP3* and a variant of *LC3A*, were previously reported respectively in BC, leukemia, astrocytoma, colorectal cancer and esophageal carcinoma and these data demonstrated that this hypermethylation was correlated to tumor grade [15–19]. Moreover, inhibition of the HMT EZH2 or G9a promotes autophagy in cancer cells while the use of HDACi gave contradictory results on autophagy levels in cancer cells [12, 40].

Despite a high homology between the different members of the GABARAP family (GABARAPL1 shows 87 % identity with GABARAP and 61 % with GABARAPL2), these proteins are differentially expressed during development and in adult tissues (for review see [41]) and these proteins have been described to be involved in several molecular pathways, including receptor transport and autophagy. Previous studies revealed that GABARAP and GABARAPL1 may have redundant functions in transport but experiments of invalidation/overexpression of GABARAP and GABARAPL1 showed that both are required for autophagy [42, 43]. *GABARAPL1* expression has been described to be decreased in cancer cell lines. MCF-7 cells stably expressing exogenous Flag-GABARAPL1-6His presents a significantly reduced proliferation rate compared to control cells [6]. Other studies described that a high expression of this gene is associated with a positive outcome in metastatic BC [5, 6] while a low expression of *GABARAPL1* was also associated with a poor outcome in kidney carcinoma patients [44]. Our results presented in this study confirmed these data by showing an inverse correlation between *GABARAPL1* expression and BC grade (Fig. 1). While *GABARAP* and *GABARAPL2* expression are also reduced in the tumor samples, this decrease was not significant. Similar differences were observed between non tumoral MCF-10A cells and MCF-7 BC cells since we showed a significant decrease of *GABARAP* and *GABARAPL2* expression in MCF-7 cells but the highest decrease was observed for *GABARAPL1*. Based on previous data reporting an epigenetic regulation of *BECN*-*1* in BC and our data showing a deregulation of DNA methylation in our BC samples (Fig. 2a, and Additional file 1: Figure S1), we hypothesized that the loss of *GABARAPL1* expression in BC might be linked to epigenetic modifications. Indeed, DNA methylation dependent gene silencing is frequent in BC and is highly related to BC tumor genotypes (*e.g*. hypermethylation of *ESR1* in ERα negative patients), and may be essential to determine the good treatment [45]. We report here that the *GABARAPL1* gene is highly methylated in both–600 (MC1) and +200 (MC2) promoter regions but the 5′-UTR +200 region presents the more significant difference between non tumoral tissues/cells promoter regions and tumoral/cancer cells since this region is poorly methylated in normal tissue and frequently methylated in tumors (Fig. 2). As described in previous studies, a correlation between local DNA methylation and histone modifications is frequently observed in epigenetic-mediated gene silencing [46, 47]. Our results confirm these observations, since we observed that the promoter of *GABARAPL1* presented a high level of methylated DNA and deacetylated histone H3 (Fig. 3). On the other hand, DNA methylation seemed poorly involved in *GABARAP* and *GABARAPL2* expression while the inhibition of HDACs by TSA increased H3 acetylation and increased *GABARAP* and *GABARAPL2* expression (Figs. 3 and 4).

Since the control of transcription factor accessibility in the region close to the transcription initiation site is often crucial for gene expression, we wondered whether it might be important for the regulation of *GABARAPL1* expression. According to transcription binding site prediction softwares, our data indeed revealed that *GABARAPL1* expression is controlled by CREB-1 and that inhibition of epigenetic repressive marks increased CREB-1-recruitment on the *GABARAPL1* promoter. Since CREB-1 has been previously involved in the regulation of autophagy, the role of this transcriptional factor may be crucial in this process. Indeed, neuron protection mediated by CREB-1 activation and associated with an increase of BECN-1 and LC3 expression, was observed following rapamycin administration in ischemic neonatal rats [48]. On the opposite, CREB-1 activation together with mTOR inhibitors has also been described to potentiate chemotherapies in renal cancers [49]. Nevertheless, the mechanism by which CREB-1 can regulate autophagy and pro-survival signals in cancer cells will require further studies.

## Conclusion

The current use of epigenetic drugs in clinical trials provides new options for personalized adjuvant therapies in cancers. Indeed, HDACi, such as Entinostat or valproic acid efficiently increase ERα expression in ER-BC tumors and considerably improve the efficiency of anti-estrogen signaling therapies [50, 51]. 5-aza-CdR treatment has also been previously reported to increase autophagy by inducing LC3B-II in myeloid cells and myeloid cells resistant to 5-aza-CdR presented an increase of basal autophagy [52]. An increase of GABARAP family protein expression, following 5-aza-CdR/TSA treatment, might at least partly explain this increase of autophagy levels. Indeed, GABARAP/GABARAPL1 overexpression in BC has been described to decrease cell proliferation and tumorigenesis in nude mice [53, 54]. All these data strongly support the idea that autophagy regulation may be a focal point for the design of combined anti-cancer therapies in the future. Mahalingam *et al*. recently proposed the evaluation of a combination of HDACi (vorinostat) and autophagy inhibitor (hydroxychloroquine) in a phase I study in patients with solid tumors [55]. However, identification of the molecular mechanisms governing gene silencing in autophagy impairment in cancer will definitely help to develop future specific drugs, and decrease the important side effects. Indeed, while DNMTi efficiently restore *ER*α or *BECN*-*1* expression in methylated tumors, these compounds also reduce global DNA methylation and local methylation [56]. DNMTi also strongly induce metalloproteinase expression in lymphoma and pancreatic cancers suggesting an increase of metastatic potential [57]. Similarly, urokinase, a marker of invasiveness associated with the most aggressive BC and with prostate cancers is increased after DNMTi treatment [58, 59].. In agreement with previous studies on different autophagy related-genes, our work demonstrated for the first time that the *GABARAP* family genes, and particularly *GABARAPL1*, are regulated by epigenetic modifications in BC and that epigenetic inhibitors might be used in combination with classical anti-chemotherapeutive drugs for futures anti-cancer therapies.

## Additional files

Additional file 1: Figure S1. *XIST* and *GAPDH* methylation status in BC. *XIST* and *GAPDH* methylation was quantified using methylCollector kit. NT: non tumoral, ER +/−: status of expression of estrogen receptor α, PR+/−: status of expression of progesterone receptor, HER+/−: status of expression of Human epidermal growth factor receptor. White : absence of signal ; black : signal of methylation. (TIFF 77 kb)
Additional file 2: Figure S2. Effects of *5*-*aza*-*CdR*/*TSA treatment on autophagy and cell proliferation*. Increase of both cytosolic LC3-I and autophagosome associated LC3-II forms detected by western blotting using lysates of MCF-7 cells treated with 5-aza-CdR/TSA (antibody anti-LC3: L8918, Sigma-Aldrich). (B) Increase of the number of cells presenting vesicles in GFP-LC3 positive MCF-7 cells transfected with GFP-LC3 and treated with 5-aza-CdR or TSA. (C) A decrease of cell proliferation was observed in MCF-7 cells treated with 5-aza-CdR/TSA using the crystal violet/acid acetic method as previously described [61]. (D) A decrease of clonogenecity was observed in MCF-7 treated with 5-aza-CdR/TSA using the crystal violet method as previously described [62]. (TIFF 122 kb)

## Abbreviations

ATG: Autophagy-related protein
BECN-1: BECLIN-1
BC: Breast Cancer
ChIP: Chromatin immunoprecipitation
CREB-1: cAMP response element binding protein
DNMT: DNA methyl transferase
DNMTi: DNMT inhibitor
ER: Estrogen receptor
GABARAP: GABA_A_-receptor associated protein
GABARAPL1/GEC1: GABARAP like protein 1/Glandular Epithelial Cells 1
GL1: GABARAPL1
GABARAPL2/GATE-16: GABARAP like protein 2/golgi-associated ATPase enhancer of 16 kDa
HDAC: Histone deacetylase
HDACi: HDAC inhibitor
HDM: Histone demethylase
HER: Human epidermal growth factor
hMOF: Human ortholog of drosophila males absent in the first
HMT: Histone methyl transferase
IF: Immunofluorescence
MAP-LC3: Microtubule associated protein-Light chain 3
PBS: Phosphate buffer saline
PBS-T: PBS-triton-×100
PFA: Paraformaldehyde
PR: Progesterone receptor
PVDF: Polyvinylidene difluoride
SDS: Sodium dodecylsulfate
TBS-T: Tris buffer saline tween 20
TSA: Trichostatin A
5aza-CdR: 5-aza-deoxycytidine
