# Supplementary figures and images for: The autophagy GABARAPL1 gene is epigenetically regulated in breast cancer models
Source: BMC Cancer. 2015 Oct 17;15:729. doi: 10.1186/s12885-015-1761-4 (PMC4609056; doi:10.1186/s12885-015-1761-4)

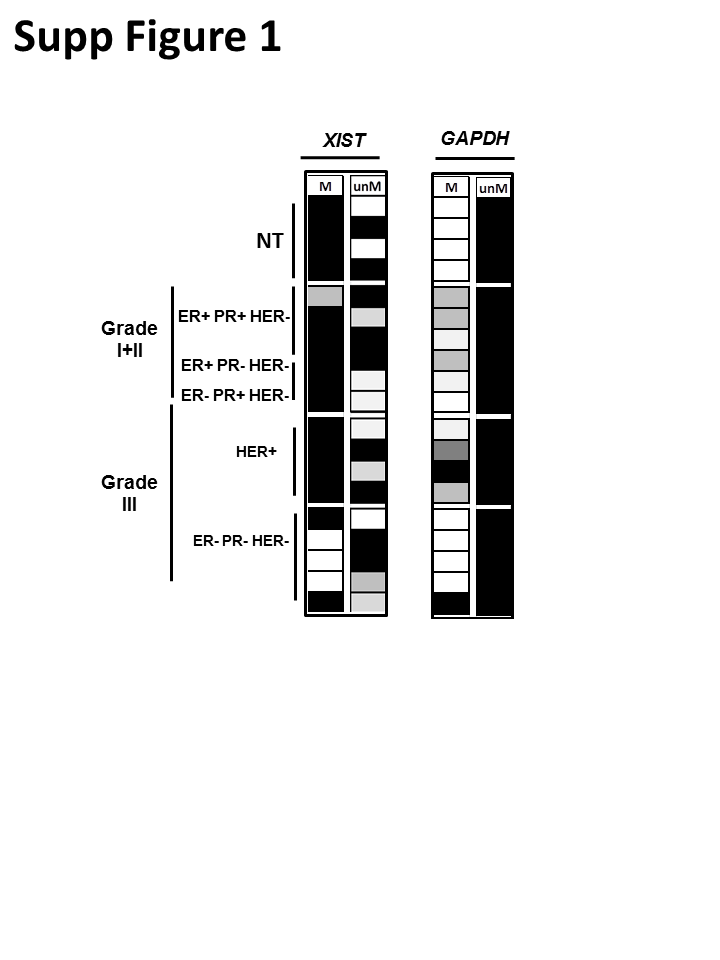

Supplement: Additional file 1: Figure S1. — XIST and GAPDH methylation status in BC. XIST and GAPDH methylation was quantified using methylCollector kit. NT: non tumoral, ER +/−: status of expression of estrogen receptor α, PR+/−: status of expression of progesterone receptor, HER+/−: status of expression of Human epidermal growth factor receptor. White : absence of signal ; black : signal of methylation. (TIFF 77 kb) [file 12885_2015_1761_MOESM1_ESM.tiff]

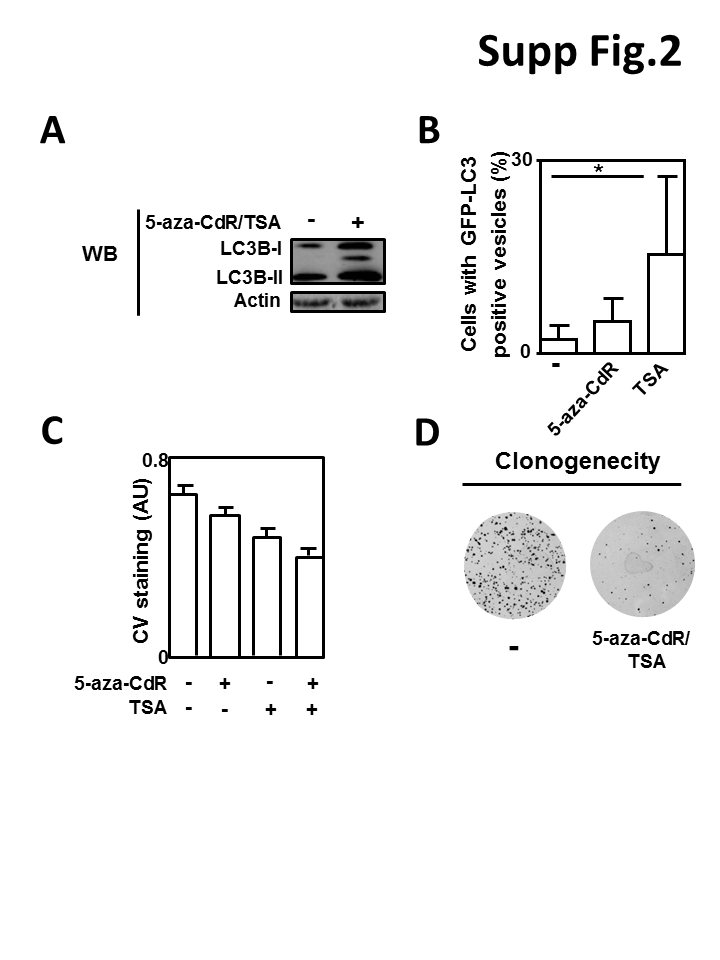

Supplement: Additional file 2: Figure S2. — Effects of 5-aza-CdR/TSA treatment on autophagy and cell proliferation. Increase of both cytosolic LC3-I and autophagosome associated LC3-II forms detected by western blotting using lysates of MCF-7 cells treated with 5-aza-CdR/TSA (antibody anti-LC3: L8918, Sigma-Aldrich). (B) Increase of the number of cells presenting vesicles in GFP-LC3 positive MCF-7 cells transfected with GFP-LC3 and treated with 5-aza-CdR or TSA. (C) A decrease of cell proliferation was observed in MCF-7 cells treated with 5-aza-CdR/TSA using the crystal violet/acid acetic method as previously described [61]. (D) A decrease of clonogenecity was observed in MCF-7 treated with 5-aza-CdR/TSA using the crystal violet method as previously described [62]. (TIFF 122 kb) [file 12885_2015_1761_MOESM2_ESM.tiff]
